# Metabolic Markers Demonstrate the Heterogeneity of Walking Ability in Non-Disabled Community-Dwelling Older Adults

**DOI:** 10.3390/metabo15050334

**Published:** 2025-05-19

**Authors:** Shanshan Yao, Ziling Mao, Megan M. Marron, Eleanor M. Simonsick, Venkatesh L. Murthy, Ravi V. Shah, Anne B. Newman

**Affiliations:** 1 Department of Epidemiology, School of Public Health, University of Pittsburgh, Pittsburgh, PA 15261, USA; shanshanyao@pitt.edu (S.Y.); zim12@pitt.edu (Z.M.); mmm133@pitt.edu (M.M.M.); 2 Intramural Research Program, National Institute on Aging, Baltimore, MD 21224, USA; simonsickel@grc.nia.nih.gov; 3 Department of Medicine and Radiology, University of Michigan, Ann Arbor, MI 48109, USA; 4 Department of Cardiovascular Medicine, Vanderbilt University Medical Center, Nashville, TN 37232, USA

**Keywords:** mobility, metabolomics, energy production

## Abstract

**Background**: Walking ability is important for the quality of life of older adults. A self-reported walking ability index (WAI) covering the difficulty and ease of walking captures a broader spectrum of walking ability in healthy older persons. **Methods**: Using metabolomics in the Health, Aging and Body Composition study, we identified Year 2 metabolites cross-sectionally and longitudinally related to WAI (0–9, higher scores indicate better walking ability) using probabilistic index models and multinomial logistic models, respectively. **Results**: Among 2334 participants (mean age 74.6 years, 51% women, 37% Black), 27% scored 0–5, 36% scored 6–8, and 37% scored 9 at Year 2. Over 4 years, 52% maintained a stable WAI, 6% improved, while 42% declined (22% 1–2 points and 20% >2 points decline). We identified 81 metabolites significantly associated with both poorer concurrent WAI and faster decline, including higher acylcarnitine species, shorter-chain saturated diglycerides and triglycerides, and TCA cycle intermediates (cis-aconitic, fumaric, and malic acids), and lower phospholipids levels. Eighteen additional metabolites were only associated with faster WAI decline: higher short-chain saturated triglycerides and energy metabolism markers (ATP/ADP/AMP) and lower margaric acid and glycine levels. Notably, those with improved WAI, despite poorer baseline WAI and lifestyles, showed more favorable metabolic profiles than others. **Conclusions**: Metabolites linked to the TCA cycle and energy metabolism, as well as inflammation and protein catabolism, were related to mobility function. Some metabolites might be particularly important for the early detection of older adults at risk of mobility decline. Metabolic profiles may also help identify older individuals (i.e., with improving WAI) with greater metabolic resilience to lifestyle risk factors and health conditions.

## 1. Introduction

Walking ability is a reflection of health status in older adults and is important for healthy aging and quality of life. Approximately 30% of older adults aged 65 years and older report difficulty in walking a quarter of a mile, with this percentage rising to over 57% in those aged 85 years and older [1]. Impaired walking ability is associated with multiple adverse health outcomes, including increased risk of falls, hospitalization, and eventually mortality [2,3].

Given the multifaceted nature of mobility decline, understanding its biological underpinnings is crucial. Metabolomics, the comprehensive study of endogenous and exogenous metabolites [4], provides a potentially useful tool to improve our understanding of these pathways. For instance, previous reports have identified several metabolite species, including triacylglycerols and phospholipids, that were associated with walking ability [5,6,7]. Despite these findings, previous evidence was mostly limited to gait speed, an established performance-based measure for assessing the walking capacity of older adults [8,9,10]. While gait speed is a validated measure of walking ability, it typically requires in-person assessment, which is not always possible in settings such as surveys. In contrast, traditional self-report measures (e.g., activity of daily living), widely used in surveys, often focus on the inability or difficulty to perform a variety of functions. Therefore, they may fail to capture subtle differences in walking ability, especially among those with no or minimal impairments. To broaden the assessment of self-reported walking ability, one approach is to incorporate questions covering higher levels of walking activities. In the Health, Aging and Body Composition (Health ABC) study, we previously developed a walking ability index (WAI) to assess both difficulty and ease in walking tasks, capturing a broader spectrum of walking ability in healthy non-disabled community-dwelling older adults [11,12].

Building on this approach, our study aims to utilize metabolomics data from the Health ABC study to identify metabolites and metabolic pathways that are cross-sectionally and longitudinally associated with walking ability, as measured by WAI. Such knowledge can offer insights into the biological mechanisms underlying mobility decline and may reveal new intervention targets to prevent walking impairment and its related adverse consequences.

## 2. Materials and Methods

### 2.1. Population

The Health ABC study [13] is a longitudinal cohort of 3075 community-dwelling older adults, including White men, White women, Black men, and Black women, aged 70–79 at baseline (i.e., Year 1). Participants were recruited from Pittsburgh, PA and Memphis, TN, from March 1997 to April 1998, of whom 1584 (52%) were women and 1281 (42%) self-identified as Black. Eligible participants reported no difficulty walking a quarter of a mile, climbing one flight of stairs without resting, or performing basic activities of daily living without an assistive device such as a cane or a walker. They did not receive active treatment for cancer during the past three years and did not have plans to leave the area within three years of enrollment. The protocol was approved by the institutional review boards at the two field centers and the coordinating center. All participants provided written informed consent.

Among the 3075 participants, 2464 participants had high-quality fasting plasma samples collected at the Year 2 visit (1998/1999) and were included in the metabolomics program [14]. Among them, 2334 participants had walking ability assessed at Year 2 and at two or more follow-up visits through the Year 6 visit (2002/2003) and were included in this study.

### 2.2. Walking Ability Index (WAI)

In the Health ABC study, self-reported walking ability was assessed every 6 months at annual clinic visits and semi-annual telephone interviews. WAI score was determined based on a series of questions on walking for ¼ mile and 1 mile. Questions on the ability to walk for ¼ mile began with, “Because of a health or physical problem, do you have any difficulty walking ¼ mile, that is about 2 or 3 blocks”? Those who reported difficulty were further asked whether they had a little, some, or a lot of difficulty or were unable to walk. Participants who denied having difficulty were further asked how easy it was for them to walk ¼ mile–very, somewhat, or not so easy. Followed by that, responses on the ease of walking for one mile were collected. Participants were then asked whether they have any difficulty walking one mile and the ease of walking one mile if no difficulty was reported. Responses were combined to create a WAI ranging from 0 to 9, where 0 represents unable to walk ¼ mile and 9 indicates that walking one mile is very easy [15].

Based on repeated WAI measurements, we utilized a mixed-effect model to estimate the individual-specific annual change of the Health ABC participants over four years while adjusting for Year 2 walking ability, age, and race–sex groups. Based on the estimated change over 4 years (Year 2 to Year 6), we then grouped the participants into “Fast decline” (>2-point decline, which was defined as a meaningful decline previously [15]), “Slow decline” (2-point ≥ decline > 1-point), “Stable” (≤1-point change), and “Improve” (>1-point improvement).

### 2.3. Metabolomics

Metabolites were measured in plasma collected at the Year 2 visit after an overnight fast of ≥ 8 h using liquid chromatography–mass spectrometry (LC-MS) methods. Fasting blood samples were collected via antecubital venipuncture in the morning, centrifuged within 15 min at 4 °C for 10 min, and frozen at −80 °C within 30 min of aliquot. Samples had not been previously thawed until metabolite profiling. Metabolite profiling was performed at the Broad Institute (Cambridge, MA, USA). Four different LC-MS methodologies, including (1) positive ion mode MS detection for polar metabolite profiling, (2) negative ion mode MS detection for polar metabolite profiling, (3) lipid profiling method, and (4) intermediate polarity profiling method, were employed to quantify metabolites [16,17]. Five prostaglandin compounds, including 15R-15-methyl PGA2, 15S-15-methyl PGD2, 15S-15-methyl PGE1, 15S-15-methyl PGE2, and 15R-15-methyl PGF2a, were used as internal standards for quality control. Details of the metabolite profiling have been published elsewhere [16,17]. Some metabolites were measured on more than one platform, which yielded 613 (520 unique) known metabolites, including duplicates. In this analysis, we included 500 (442 unique) known metabolites, which were quantified in ≥90% of participants and had a coefficient of variation ≤ 10% [14]. When duplicates of lipids or lipid-like molecules correlated with weight change groups, we presented data from the lipid profiling method when it was available. For other metabolite duplicates, we report findings from the platform demonstrating the lowest coefficient of variation. Missing values for metabolites were assumed to be below detectable limits and were imputed as 50% of the lowest value detected for the respective metabolite [14]. Metabolite values were log-transformed and standardized for further analyses.

### 2.4. Covariates

We investigated the impact of potential confounders, including demographics, lifestyle, and health conditions, collected at baseline or Year 2 visits on identified metabolite associations with walking ability. Age was self-reported at the Year 2 visit. Sex, race, highest level of education, smoking habits, and sleep duration were collected via self-report at baseline (i.e., Year 1) visit. Appetite was self-reported at Year 2 and was categorized into “Very good”, “Good”, and “Moderate to poor or fluctuating”. The Healthy Eating Index (HEI) score based on a 108-item interviewer-administered food frequency questionnaire was calculated to represent diet quality [18,19]. Physical activity was defined by a calculated energy expenditure (kcal/kg/week) in walking and climbing stairs at the Year 2 visit. Body mass index (BMI) was calculated using height and weight measured at the Year 2 visit and was categorized into < 25 kg/m^2^, 25–30 kg/m^2^, and ≥ 30 kg/m^2^ to address nonlinear relationships. Blood biomarkers of inflammation, interleukin-6 (IL-6), and C-reactive protein (CRP) were measured using frozen stored serum collected after an overnight fast at the Year 2 visit [20]. Renal function biomarkers, including serum cystatin C and creatinine, were measured at baseline. History or the presence of cardiovascular disease, hypertension, diabetes, and cancer were determined based on participants’ self-report of a physician diagnosis. Hypertension and diabetes were also determined based on medication reported at Year 2. The presence of peripheral artery disease, osteoporosis, depression, and pulmonary disease was collected at Year 1. The number of prescription medications used was collected through a medication inventory at the Year 2 clinic visit.

### 2.5. Statistical Analysis

We summarized baseline characteristics of the study sample using means (standard deviation, SD) or medians [Q1, Q3] for continuous variables and frequency (proportions) for categorical variables. The statistical differences in participants’ characteristics across different WAI groups were tested by one-way analysis of variance or Kruskal–Wallis rank sum test for continuous variables and Pearson’s Chi-squared test for categorical variables.

We then conducted individual metabolite analysis to explore Year 2 metabolites cross-sectionally associated with Year 2 WAI after adjusting for age, race–sex group, and study site using a semiparametric model–the Probabilistic Index Model (PIM) with a logit link [21]. Given the discrete nature and skewed distribution of the WAI in our study sample, an ordinary linear regression model was not appropriate for our purpose. The PIM was determined appropriate because it is an extension of the Wilcoxon–Mann–Whitney test, which can handle both continuous and discrete response variables and does not require a normality assumption [21,22]. The PIM was conducted using the “*pim*” R package. For our study, the PIM estimated the adjusted odds of a participant with a 1-SD higher value of the metabolite having a higher WAI score than a participant with a lower value of that metabolite [21,22]. The generalized odds ratio [exponentiate of beta coefficient, exp(β)] is interpreted as “*For a pair of individuals who differ by 1 SD in their metabolite levels, the odds that the individual with higher metabolite level has higher WAI than the individual with lower metabolite level is exp(β), controlling for age, race-sex group, and study site*”.

Next, we investigated the prospective relationships between each Year 2 metabolite and WAI change groups from Year 2 to Year 6 relative to the “Stable” group using multinomial logistic regression while adjusting for age, race–sex group, study site, and baseline WAI. The odds ratio [exponentiate of beta coefficient, exp(β)] is interpreted as “*Individuals with 1 SD higher metabolite level have exp(β) times the odds of being in a specific group (Improve/Slow Decline/Fast Decline) versus being in the Stable group compared to those with lower metabolite level, controlling for age, race-sex group, study site, and baseline WAI*”.

After identifying metabolites associated with both baseline WAI and WAI change, we determined whether these metabolite associations differed by race and sex subgroups (Black men, Black women, White men, and White women). Moreover, we investigated whether the observed metabolite associations would be attenuated after further adjusting for known risk factors associated with WAI in older adults, including smoking, self-reported appetite, dietary quality (HEI), physical activity, BMI category, inflammation biomarkers (IL-6 and CRP), renal function (cystatin C and creatinine), chronic disease history, number of prescription medications, and the combination of aforementioned risk factors. We examined the extent to which these variables explained the minimally adjusted associations between metabolites and WAI using percent attenuation calculated as 100 × ( β1 − β2)/β1, where β1 is the minimally adjusted beta coefficient between metabolite values and WAI, while β2 is the beta coefficient after further adjusting for other risk factors.

To account for multiple comparisons in individual metabolite associations, we used the Benjamini–Hochberg correction with a 5% false discovery rate (FDR) [23]. All statistical analyses were conducted using R software version 4.2.1 (R Foundation for Statistical Computing).

Metabolites that were significantly associated with WAI after adjusting for age, sex–race, study site, and multiple comparisons were further examined in a pathway analysis using MetaboAnalyst version 6.0 on 12 March 2025 [24]. The pathway analysis compared the targeted set of metabolites associated with WAI against sets of metabolites known to be involved in metabolic pathways and tested whether the overlap was more than expected by chance using Fisher’s exact test. We conducted the pathway analysis using two sets of reference metabolome: first, all metabolites available in the MetaboAnalyst pathway library and second, only the metabolites measured in the Health ABC study where only 264 metabolites were matched. The second approach, while more conservative, helps avoid biases due to unmeasured metabolites from the specific technology. Impact scores indicate the impact on the pathway if the levels of identified metabolites involved in certain pathways were altered. The possible range of an impact score is from zero to one, with a higher score indicating that the metabolites involved have a greater impact on the pathway.

## 3. Results

### 3.1. Participant Characteristics

In our study of 2334 older adults (mean age 74.6 years, SD 2.9), 51% were women, and 37% were Black. Participants with WAI scores 0–5 (n = 623, 27%) reported either “having difficulty” walking one mile and finding a quarter-mile walk “not very easy” or found walking both distances “not that easy”. Compared to those with the best walking ability (WAI score 9–“very easy” walking one mile; n = 860, 37%), these participants were older, more likely to be women and Black, and had lower education levels. They were also more likely to be a smoker, obese, had poor appetite, poor diet quality, low physical activity, a chronic disease, more medication use, and higher inflammation levels. In addition, they were more likely to report a WAI decline during the following 4 years (Table 1).

**Figure 1:**
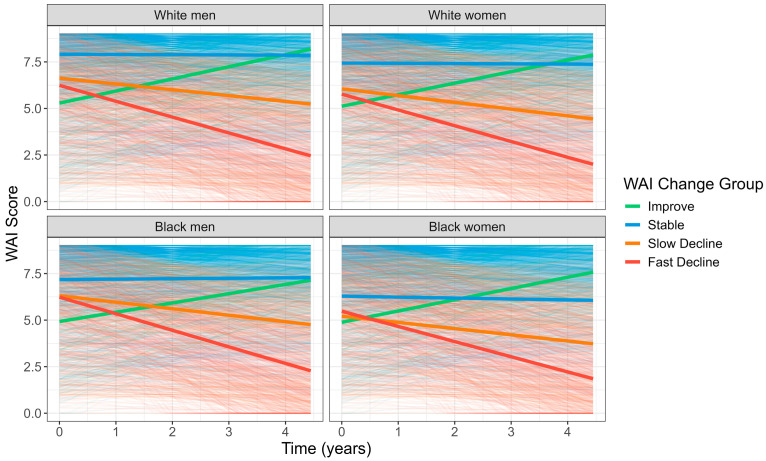
Trajectory of walking ability index (WAI) over time in participants from the Health, Aging and Body Composition Study by WAI change groups across race–sex groups.

Over a four-year period (Years 2–6), 52% (n = 1203) maintained stable walking ability, while 42% declined: 22% (n = 520) slowly and 20% (n = 463) rapidly. The mean WAI trajectories were similar across race–sex groups, with all four WAI change groups showing a consistent rate of change, except White participants in the “Stable” group showed better (higher) initial WAI scores compared to Black participants (Figure 1). Compared to the “Stable” group, both declining groups had slightly lower initial WAI, had more women, Black participants, and lower education levels. They also had a higher prevalence of smoking, obesity, poor appetite, and chronic diseases, had more frequent hospitalization during the study period, and had poorer diet quality, lower physical activity, more medication use, and higher inflammation levels. The “Improve” group had the lowest initial WAI among the four groups, but they showed better health profiles than the two “declining” groups and worse health than the “Stable” group (Table 2).

### 3.2. Cross-Sectional Metabolite Associations

**Figure 2:**
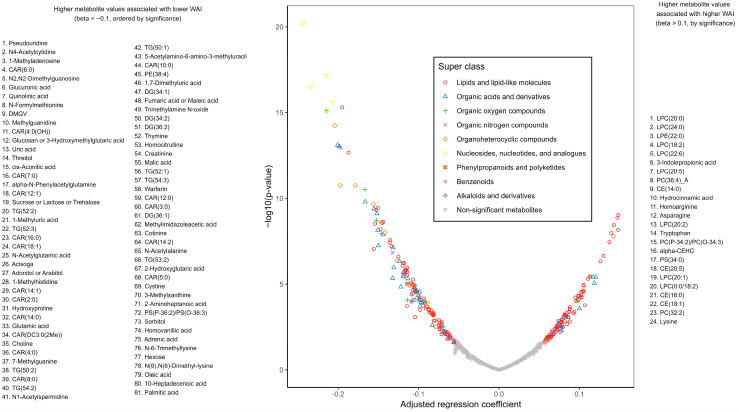
Cross-sectional metabolite associations with Year 2 walking ability index (WAI) after adjusting for age, race–sex groups, and study site in participants from the Health, Aging and Body Composition Study, from probabilistic index models.

There were 143 metabolites cross-sectionally associated with worse (lower) WAI at the significance level FDR < 0.05 after adjusting for age, race–sex groups, and study site, of which 81 metabolites showed a stronger negative association (beta coefficient < −0.1). Among these 81 metabolites, 17 were acylcarnitines, 11 were triglycerides (TGs, 50–54 carbon atoms and ≤3 double bonds) and diglycerides (DGs, 34–36 carbon atoms and ≤2 double bonds), 4 were fatty acids (10-heptadecenoic, adrenic, oleic, and palmitic acids), 12 were amino acids and their derivatives (e.g., N-acetylalanine, N-formylmethionine, cystine), 6 carbohydrates (e.g., glucuronic acid, sucrose/lactose/trehalose), 6 were purines and purine derivatives, and 25 metabolites of other classes. Ninety-two metabolites were associated with better (higher) WAI, and twenty-four of them had a beta coefficient >0.1. Among the 24 metabolites, 12 were phospholipids (8 lysophosphatidylcholines, e.g., LPC(18:2), LPC(20:0), LPC(24:0) and 4 other phospholipids), 4 were cholesterol esters (e.g., CE(18:1)), 4 were amino acids (asparagine, homoarginine, lysine, tryptophan), and 4 were other metabolites (Figure 2). There was no significant interaction between cross-sectional metabolite associations and sex and race.

### 3.3. Longitudinal Metabolite Associations

**Figure 3:**
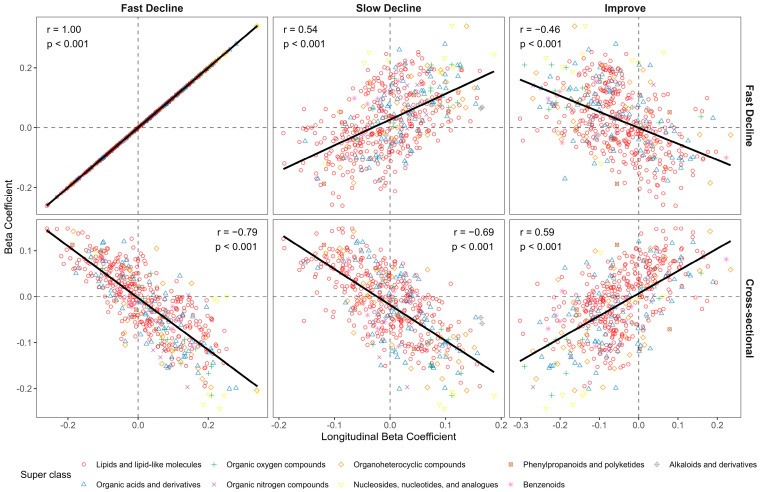
Metabolite associations with Year 2 walking ability index (WAI) and WAI change groups in participants from the Health, Aging and Body Composition Study.

The longitudinal analysis showed that while no metabolites reached statistical significance (FDR < 0.05) for the “Improve” and “Slow decline” groups, their metabolic patterns aligned with those seen in the “Fast decline” group. Specifically, the metabolite associations for “Fast decline” showed a positive correlation with those for “Slow decline” (Pearson correlation r = 0.54) and a negative correlation with those for “Improve” (r = −0.46) (Figure 3).

**Figure 4:**
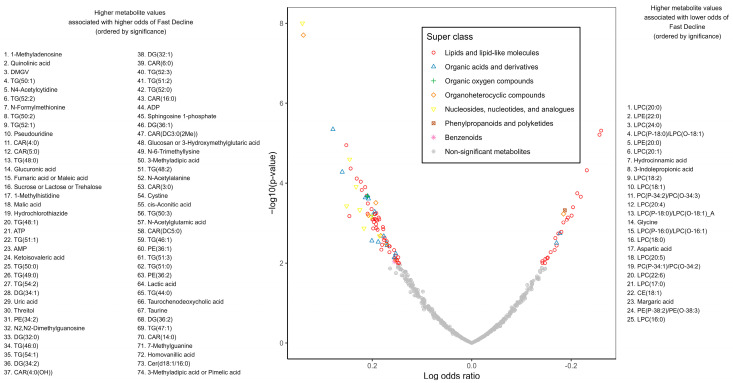
Longitudinal metabolite associations with fast decline in walking ability index (WAI) compared to maintaining a stable WAI from Year 2 to Year 4 after adjusting for age, race–sex groups, and study site in participants from the Health, Aging and Body Composition Study, from multinomial logistic regression models.

Seventy-four metabolites were significantly associated with higher odds of experiencing a fast decline in WAI after adjusting for age, race–sex groups, study site, and initial WAI: 28 TGs (44–56 carbon atoms and ≤3 double bonds) and diglycerides (32–34 carbon atoms and ≤1 double bond), 9 acylcarnitines, 6 amino acids and derivatives (e.g., N-acetylalanine, N-formylmethionine, cystine), 3 phosphatidylethanolamines, 3 purine nucleotides (ADP, AMP, ATP), 8 organic acids, 4 carbohydrates (e.g., glucuronic acid, sucrose/lactose/trehalose), and 13 other metabolites. Additionally, 25 metabolites were associated with lower odds of experiencing a fast WAI decline: 19 phospholipids (15 lysophosphatidylcholines e.g., LPC(18:2), LPC(20:0), LPC(24:0), 4 other phospholipids), 2 fatty acids (hydrocinnamic acid, Margaric acid), 1 cholesterol ester (CE(18:1)), and 3 amino acids and derivatives (glycine, aspartic acid, 3-indolepropionic acid) (Figure 4). There was no significant interaction between cross-sectional metabolite associations and sex and race.

In addition, the cross-sectional and longitudinal metabolite associations with WAI were consistent in relative magnitude and direction (Figure 3). That is, metabolites associated with better concurrent WAI were also associated with higher odds of improving (r = 0.59) and lower odds of declining WAI (r = −0.69 for “Slow decline” and −0.79 for “Fast decline”) over 4 years subsequently. Among the 99 metabolites significantly associated with subsequent fast decline in WAI, 81 (81%) were also significantly related to concurrent WAI. The remaining 18 (19%) metabolites that were only longitudinally but not cross-sectionally associated with WAI included 9 TGs (44–51 carbon atoms and ≤2 double bonds), 1 phosphosphingolipid (sphingosine 1-phosphate), 1 bile acid (taurochenodeoxycholic acid), 3 purine ribonucleotides (AMP, ADP, ATP), and 2 organic acids (ketoisovaleric acid and taurine) that were associated with higher odds of fast WAI decline, as well as 1 long-chain fatty acid (margaric acid) and 1 amino acid (glycine) that were associated with lower odds of fast WAI decline.

### 3.4. Investigation of Potential Confounders

**Figure 5:**
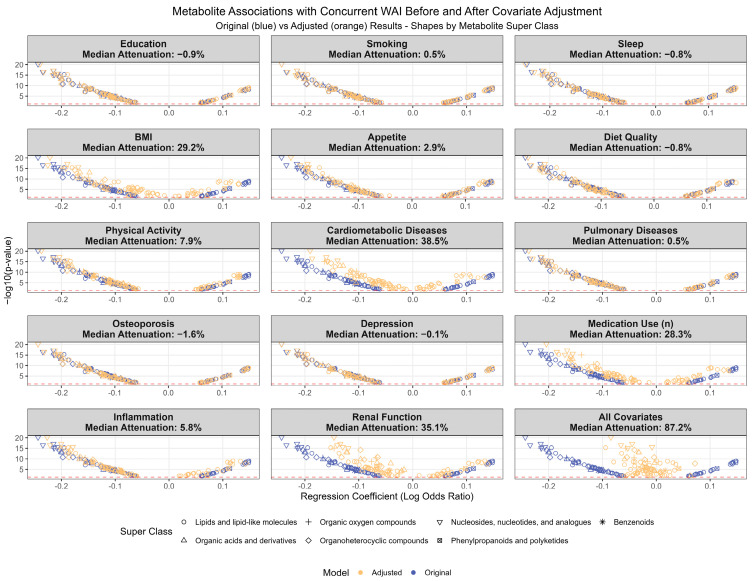
Cross-sectional associations between 81 metabolites and concurrent walking ability index (WAI) before and after adjusting for potential confounders in addition to age, race–sex groups, and study site in participants from the Health, Aging and Body Composition Study.

Among the 81 metabolites that were consistently related to both concurrent WAI and fast WAI decline over time, we examined how adjusting for potential confounders might attenuate the metabolite associations. In addition to demographics, further adjusting for the presence of cardiometabolic diseases (including Year 2 cardiovascular disease, hypertension, diabetes, and Year 1 peripheral artery disease) and poorer renal function (cystatin C and creatinine) each attenuated half of the cross-sectional metabolite associations by >35%. Higher Year 2 BMI and higher number of prescription medication use also attenuated many associations. After full adjustment for all included covariates, 80 of the 81 metabolite associations were attenuated by at least 48%, leaving aspartic acid as the only metabolite with a robust association with WAI (beta coefficient ~0.06 before and after adjustment). However, upon correcting for multiple comparisons, only CAR(6:0) remained significantly associated with lower odds of a better concurrent WAI (Figure 5).

**Figure 6:**
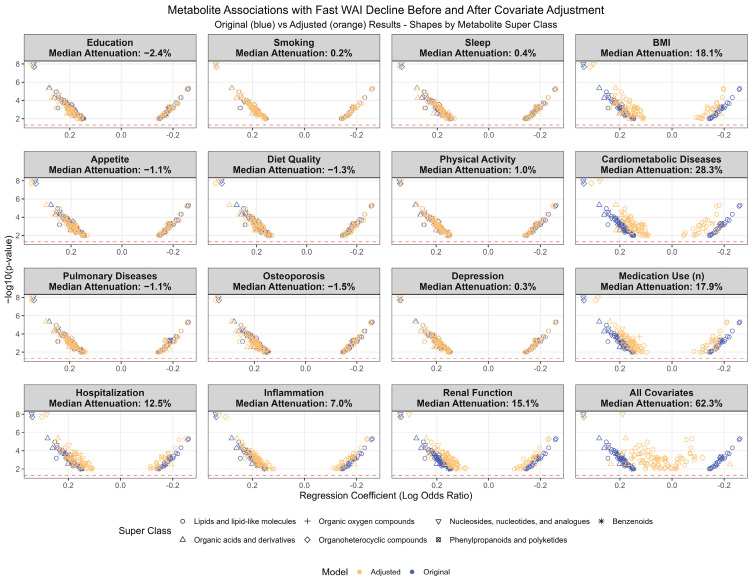
Longitudinal associations between 81 metabolites and fast decline in walking ability index (WAI) over four years before and after adjusting for potential confounders in addition to age, race–sex groups, study site, and initial WAI in participants from the Health, Aging and Body Composition Study.

Similarly, the longitudinal metabolite associations with fast WAI decline were more substantially attenuated by cardiometabolic diseases, renal function, BMI, and number of prescription medications used than by other risk factors. More frequent hospitalization during the study period, i.e., Year 2 to Year 6, also attenuated many metabolite associations (median attenuation 12.5%). Seven metabolite associations—including aspartic acid, N-Acetylalanine, 3-methyladipic acid, quinolinic acid, 1-methylhistatine, cystine, and CAR(DC:5:0)—were relatively robust, exhibiting < 20% attenuation in the fully adjusted model. Only aspartic acid and quinolinic acid remained statistically significant after multiple comparison adjustments (FDR < 0.05) (Figure 6).

### 3.5. Pathway Analysis

We then performed pathway analysis based on 81 metabolites related to both concurrent WAI and fast WAI decline, as well as on 18 metabolites related to fast WAI decline only. As shown in Table 3, energy metabolism pathways including citrate cycle (TCA cycle) and pyruvate metabolism, amino acid processing including arginine, histidine, and alanine/aspartate/glutamate metabolism, and nicotinate metabolism, were associated with both lower current WAI and fast WAI decline. In addition, metabolites exclusively associated with fast WAI decline were primarily involved in bile acid biosynthesis, purine metabolism (including AMP, ADP, and ATP), one-carbon metabolism, and taurine pathways.

## 4. Discussion

Among 2334 older Black and White men and women who had no reported mobility limitation at baseline, we identified consistent metabolite associations that demonstrated heterogeneity in both current and future walking ability change. Phospholipids were positively associated with better walking ability, while acylcarnitines, shorter-chain and more saturated TGs/DGs, and specific amino acid derivatives (e.g., N-acetylalanine, N-formylmethionine, and cystine) showed negative associations. Energy-related compounds (AMP, ADP, ATP), shorter-chain saturated TGs, and some other metabolites (e.g., sphingosine 1-phosphate and ketoisovaleric acid) were linked to a higher risk for decline, while only margaric acid and glycine were associated with mobility stability. Pathway analysis revealed that energy metabolism pathways (the TCA cycle and pyruvate metabolism) and amino acid processing were implicated by metabolites consistently related to current and future mobility, while purine metabolism emerged only for mobility decline. Furthermore, the identified metabolic signatures of walking ability in older adults appeared to be closely linked to cardiometabolic conditions, renal function, and polypharmacy.

Notably, we found consistent metabolite associations with WAI change compared to WAI stability—the metabolites related to higher odds of declining WAI were also related to lower odds of improving WAI after adjusting for initial WAI. However, we also observed that participants with improving WAI had poorer health profiles (e.g., more smokers, less physical activity, more medication use, higher inflammation level, and lower initial WAI) at the time of metabolomics profiling and more frequent hospitalization during follow-up than the “Stable” group. The observations of the health profiles and metabolites with improving WAI suggest that metabolomics may capture aspects of physiological function not fully captured by conventional risk factors. People with certain genetic or biological predispositions may have greater metabolic resilience, enabling them to maintain better metabolic profiles despite unhealthy lifestyles and health conditions. Additionally, the metabolic profiles of older adults with WAI improvement might reflect the body’s successful adaptation to stress, adverse lifestyles, or predisposing health conditions, ultimately leading to better walking ability later in life. These findings suggest that a snapshot of metabolism might better predict future health changes than traditional health measures alone. This metabolic resilience captured through metabolomics offers a promising avenue for both identifying older adults at varying risks for mobility decline and deepening our mechanistic understanding of mobility maintenance in aging populations.

Indeed, we found that the metabolite correlates of current and future WAI change were largely concordant, highlighting not only prognostic relations but potential biological mechanisms of mobility-based metabolic signatures. In this work, many of the identified metabolite associations centered on energy production, with the TCA cycle playing a central role. Pathway analysis also indicated that the TCA cycle was among the top pathways implicated by identified metabolites. For example, the higher levels of TCA cycle intermediates (i.e., cis-aconitic, fumaric, and malic acids) point to mitochondrial dysfunction, potentially affecting energy production efficiency. Some specific membrane lipids, such as lysophosphatidylcholines containing long-chain polyunsaturated fatty acids (LPC 20:4, 20:5, 22:6), showed protective associations with mobility. These membrane components are crucial for maintaining cellular function through membrane integrity, which directly impacts mitochondrial efficiency and energy production [25]. Modified nucleosides (1-methyladenosine, 7-methylguanine) have been linked to proinflammatory cytokine IL6, mitochondrial dysfunction, and frailty [26,27,28,29]. The consistency of these metabolite associations with both current and future walking ability suggests their fundamental role in mobility function rather than the consequences of mobility decline.

Specifically, a growing body of evidence points toward incomplete fatty acid oxidation during energy production. For example, higher acylcarnitines and specific saturated TGs and DGs were associated with having a poorer WAI at baseline and with a future fast decline over four years. These findings are consistent with previous reports on frailty and gait speed [6,29]. Among all TGs and DGs measured in Health ABC metabolomics (TGs with 41–60 carbon atoms and ≤10 double bonds; DGs with 32–38 carbon atoms and ≤5 double bonds), those with relatively shorter carbon chains and more saturated fatty acids were related to worse walking ability. This suggests impaired lipid metabolism that seems more closely tied to smaller TGs [25]. While direct evidence is limited, our findings are consistent with a previous report on structure-dependent relationships between TGs and diabetes risk [30], which is also linked to adiposity. Acylcarnitines related to poorer walking ability further suggest incomplete fatty acid oxidation [31]. Acylcarnitines are important carriers for fatty acids and organic acids when they are transported into the mitochondria for β-oxidation. Higher levels of acylcarnitines suggest that fatty acid intermediates in the body were not able to be fully oxidized to acetyl-CoA for the TCA cycle, leading to insufficient ATP generation [32]. Incomplete fatty acid oxidation and suboptimal ATP production may contribute to higher levels of reactive species, greater metabolic stress, and lower energy production efficiency in muscle cells, as well as promoting inflammation [33,34]. These may lead to poorer muscle function and subsequent loss of mobility [35,36].

Some amino acid species, such as aspartic acid, 3-indolepropionic acid, N-acetylalaine, N-formylmethionine, and cystine, were also related to walking ability. Among them, the relationship between aspartic acid and better walking ability was notably robust to adjustment for comprehensive risk factors. This suggests that aspartic acid may have a direct physiologic role in maintaining walking ability. Aspartic acid is known to replenish TCA cycle intermediates by converting to oxaloacetic acid, which supports robust and efficient ATP production under oxidative stress [37]. Additionally, aspartic acid is also a neurotransmitter precursor, which highlights the importance of neuromuscular signaling in sustained mobility function [37]. The protective association of 3-indolepropionic acid, a microbiota-produced tryptophan metabolite with antioxidant properties, may protect against oxidative stress in the body [38]. N-acetylalanine can increase in catabolic states or under inflammatory states and has been associated with sarcopenia [39]. Higher N-formylmethionine levels have been linked to incomplete mitochondrial fatty acid oxidation and branched-chain amino acid catabolism [40], as well as poorer physical performance and mortality [40,41]. Cystine—oxidized cysteine—but not cysteine was associated with poorer mobility, suggesting altered redox balance and excessive oxidative stress [42,43], which can impair muscle function, leading to mobility limitation and decline. These findings suggest that redox imbalance, amino acid catabolism, and excessive oxidative stress—all tied to mitochondrial function and energy production—may play important roles in muscle function maintenance and mobility.

Here, we also identified several metabolites that were only associated with fast WAI decline but not current WAI, suggesting they may be promising biomarkers for early detection and mechanistic investigation of mobility decline. Energy markers implicated in purine metabolism, i.e., ATP/ADP/AMP, demonstrated an association with higher mobility decline risk. This aligns with research establishing the critical role of the purinergic system in the control of sarcopenia and muscle homeostasis [44]. While our cross-sectional data showed no/weak associations between these energy markers and current walking ability, their emergence as predictors of future decline supports our hypothesis that energy metabolism disruption precedes observable mobility limitations. Specifically, the observed associations with AMP/ADP suggest that the ability to maintain energy homeostasis under stress conditions may determine long-term mobility outcomes. The biosynthesis of ATP occurs from ADP and AMP, and increased AMP and ADP levels signal the need for more energy production and activate pathways like glycolysis and AMP-activated protein kinase to restore ATP levels [44]. Evidence suggests that aging is linked to decreased mitochondrial ADP sensitivity in skeletal muscle, which increases mitochondrial ROS [45]. Therefore, our findings may suggest a lower ADP sensitivity predisposing to a fast mobility decline in older adults. In addition, many forms of cellular irritation, such as cell death, hypoxia, or inflammation, promote the release of ATP from the intracellular storage pool into the extracellular compartment, which induces inflammation [46,47]. Hence, the association between higher circulating ATP and mobility decline may reflect leaking energy and inflammation under cell damage or poor membrane integrity when excessive stress occurs, such as in presence of underlying diseases.

Other metabolites, such as sphingosine 1-phosphate, which is implicated in inflammatory pathways [48], and ketoisovaleric acid, which is involved in protein catabolism [49], were also associated with muscle health. In addition, we also identified two specific metabolites—margaric acid and glycine—that emerged as protective factors against future mobility decline. These protective associations provide important insights into potential preventive mechanisms against age-related mobility deterioration. Margaric acid, an odd-chain fatty acid, and a dairy fat biomarker, has been associated with lower risks of CVD and mortality [50,51]. This observation suggests that older adults who maintained a relatively stable walking ability over time might have more dairy intake than those who experienced a fast decline. This supports a beneficial role of milk fat intake or of dairy components (e.g., calcium, magnesium, vitamin D) in preserving muscle or bone health and mobility. Glycine’s protective association is in line with its role in protecting muscle cells from wasting and thus preventing mobility decline [52].

Taken together, the metabolite associations of current and future mobility were centered on pathways of energy production, with some indicating roles in protein catabolism and inflammation modulation. The consistent findings of cross-sectional and longitudinal associations reduce the possibility of reverse causality. Metabolites with consistent cross-sectional and prospective associations might be therapeutic targets, while those with only prospective associations might serve as predictive and preventive biomarkers. On the other hand, other metabolite associations (e.g., certain carbohydrates, fatty acids, and amino acids) that emerged only in cross-sectional investigations may have limited predictive power for future mobility decline, may simply reflect current health status, or may result from existing mobility limitations rather than cause them.

Furthermore, the identified metabolite associations were substantially attenuated after adjusting for the presence of cardiometabolic diseases, poorer renal function, overweight/obese, and more medication use. This suggests the roles of these risk factors as important confounders or mediators, as well as intervention targets. Hospitalization during the mobility decline period might be an important mediator in the pathway from altered metabolic states to mobility decline. This supports the potential of identified metabolic biomarkers for preventing hospitalization and its related health outcomes, including mobility decline.

This study has limitations. In this report, the WAI was based on self-report, which may be less accurate than objective measures like gait speed. However, our findings largely align with previous reports on gait speed and frailty [6,29], supporting its potential for research on mechanisms of mobility. Additionally, as a healthy older cohort, Health ABC participants were selected to have no disability at baseline, with around 37% of the participants having the highest score (WAI = 9), of which 34% showed declining mobility. Therefore, our findings may not be generalizable to other older populations (e.g., older adults with disability), and future studies are encouraged to use this tool and validate our findings. Moreover, we did not detect any significant sex- or race-specific metabolic signatures, some of which might be due to limited power. Despite these limitations, this work has several strengths, including the well-characterized biracial cohort of over 2000 older adults and the high-throughput metabolomics. Our efforts on both cross-sectional and longitudinal assessments of metabolic signatures for walking ability also strengthen the causal interpretation of our findings.

## 5. Conclusions

In conclusion, in this cohort of healthy community-dwelling older adults, the metabolic characterization of a self-reported walking ability index revealed a central role of energy production, along with lipid metabolism, protein catabolism, and inflammation, underlying mobility decline. Early interventions on lifestyles and health conditions (e.g., cardiometabolic health, renal function, and polypharmacy) may help restore balanced metabolic states while preventing or delaying mobility decline in older adults. Metabolomics is well known to be a hypothesis-generating technique and, in this study, has shown a potential to help identify subgroups with greater metabolic resilience to unhealthy lifestyles and health profiles.

## Figures and Tables

**Table 1:** Characteristics of participants from the Health, Aging and Body Composition study by Year 2 walking ability index (WAI).

		Year 2 WAI			
Characteristic	Overall N = 2334	0–5 N = 623	6–8 N = 851	9 N = 860	*p*-Value
**WAI change over 4 years**					**<0.001**
*Improve*	148 (6%)	113 (18%)	35 (4%)	0 (0%)	
*Stable*	1203 (52%)	232 (37%)	403 (47%)	568 (66%)	
*Slow Decline*	520 (22%)	166 (27%)	187 (22%)	167 (19%)	
*Fast Decline*	463 (20%)	112 (18%)	226 (27%)	125 (15%)	
**Age, year**	74.6 (2.9)	75.0 (2.9)	74.5 (2.8)	74.5 (2.8)	**<0.001**
**Race**					**<0.001**
*White*	1471 (63%)	326 (52%)	522 (61%)	623 (72%)	
*Black*	863 (37%)	297 (48%)	329 (39%)	237 (28%)	
**Sex**					**<0.001**
*Men*	1155 (49%)	253 (41%)	391 (46%)	511 (59%)	
*Women*	1179 (51%)	370 (59%)	460 (54%)	349 (41%)	
**Race and sex**					**<0.001**
*White men*	782 (34%)	146 (23%)	262 (31%)	374 (43%)	
*White women*	689 (30%)	180 (29%)	260 (31%)	249 (29%)	
*Black men*	373 (16%)	107 (17%)	129 (15%)	137 (16%)	
*Black women*	490 (21%)	190 (30%)	200 (24%)	100 (12%)	
**More than high school education**	1787 (77%)	425 (68%)	660 (78%)	702 (82%)	**<0.001**
**Smoker**	210 (9%)	72 (12%)	86 (10%)	52 (6%)	**<0.001**
**Sleep hours/night**	6.9 (1.3)	6.7 (1.5)	6.8 (1.3)	7.0 (1.2)	**0.005**
**BMI, kg/m^2^**	27.3 (4.8)	29.0 (5.4)	27.2 (4.8)	26.0 (3.9)	**<0.001**
**BMI category**					**<0.001**
*<25 kg/m^2^*	786 (34%)	147 (24%)	289 (34%)	350 (41%)	
*25–30 kg/m^2^*	977 (42%)	230 (37%)	364 (43%)	383 (45%)	
*≥30 kg/m^2^*	571 (24%)	246 (39%)	198 (23%)	127 (15%)	
**Appetite**					**<0.001**
*Very good*	970 (42%)	195 (32%)	347 (41%)	428 (50%)	
*Good*	851 (37%)	230 (38%)	300 (36%)	321 (38%)	
*Moderate to poor*	485 (21%)	182 (30%)	196 (23%)	107 (13%)	
**Healthy Eating Index, 0–100**	69.6 (12.2)	67.7 (12.4)	69.4 (12.0)	71.2 (12.1)	**<0.001**
**Physical activity (Kcal/kg/Week)**	3.0 [0.4–9.5]	0.8 [0.0–4.0]	2.4 [0.3–7.5]	7.5 [2.0–15.8]	**<0.001**
**Cardiovascular disease**	628 (27%)	224 (36%)	218 (26%)	186 (22%)	**<0.001**
**Hypertension**	1237 (53%)	421 (68%)	459 (54%)	357 (42%)	**<0.001**
**Diabetes**	911 (39%)	289 (46%)	330 (39%)	292 (34%)	**<0.001**
**Cancer**	430 (18%)	115 (18%)	141 (17%)	174 (20%)	0.15
**Peripheral artery disease**	108 (5%)	55 (9%)	36 (4%)	17 (2%)	**<0.001**
**Osteoporosis**	235 (10%)	74 (12%)	95 (11%)	66 (8%)	**0.012**
**Depression**	221 (10%)	77 (12%)	77 (9%)	67 (8%)	**0.010**
**Pulmonary disease**	261 (11%)	112 (18%)	88 (10%)	61 (7%)	**<0.001**
**Total prescription medications**	3.0 [1.0–5.0]	4.0 [2.0–6.0]	3.0 [1.0–5.0]	2.0 [1.0–4.0]	**<0.001**
**C-reactive protein (ug/mL)**	2.8 [1.2–6.2]	3.9 [1.6–7.8]	3.0 [1.3–6.3]	2.1 [1.0–4.9]	**<0.001**
**Interleukin-6 (pg/mL)**	2.3 [1.5–3.9]	2.9 [1.9–4.9]	2.3 [1.5–3.8]	2.0 [1.3–3.3]	**<0.001**
**Cystatin C (mg/dL)**	1.0 [0.9–1.1]	1.0 [0.9–1.2]	1.0 [0.9–1.1]	1.0 [0.8–1.1]	**<0.001**
**Creatinine (mg/dL)**	1.0 [0.9–1.2]	1.0 [0.9–1.2]	1.0 [0.9–1.1]	1.0 [0.9–1.1]	0.2

Note: Mean (SD), median [Q1, Q3], or frequency (%) were presented and compared using ne-way analysis of means (not assuming equal variances), Pearson’s Chi-squared test, or Kruskal–Wallis rank sum test.

**Table 2:** Characteristics of participants from the Health, Aging and Body Composition study by walking ability index (WAI) change groups.

		WAI Change Groups				
Characteristic	Overall N = 2334	Fast Decline N = 463	Slow Decline N = 520	Stable N = 1203	Improve N = 148	*p*-Value
**Initial WAI (Year 2)**	6.68 (2.56)	6.82 (1.97)	6.28 (2.79)	7.20 (2.40)	3.34 (2.04)	**<0.001**
**Age, year**	74.6 (2.9)	74.7 (2.9)	74.8 (2.8)	74.6 (2.9)	74.4 (2.8)	0.4
**Race**						**0.001**
*White*	1471 (63%)	277 (60%)	302 (58%)	804 (67%)	88 (59%)	
*Black*	863 (37%)	186 (40%)	218 (42%)	399 (33%)	60 (41%)	
**Sex**						0.051
*Men*	1155 (49%)	222 (48%)	234 (45%)	626 (52%)	73 (49%)	
*Women*	1179 (51%)	241 (52%)	286 (55%)	577 (48%)	75 (51%)	
**Race and sex**						**0.006**
*White men*	782 (34%)	139 (30%)	150 (29%)	445 (37%)	48 (32%)	
*White women*	689 (30%)	138 (30%)	152 (29%)	359 (30%)	40 (27%)	
*Black men*	373 (16%)	83 (18%)	84 (16%)	181 (15%)	25 (17%)	
*Black women*	490 (21%)	103 (22%)	134 (26%)	218 (18%)	35 (24%)	
**More than high school education**	1787 (77%)	327 (71%)	391 (75%)	957 (80%)	112 (76%)	**<0.001**
**Smoker**	210 (9.0%)	38 (8.2%)	55 (11%)	98 (8.2%)	19 (13%)	0.13
**Sleep hours/night**	6.9 (1.3)	6.8 (1.4)	6.8 (1.4)	6.9 (1.3)	6.7 (1.1)	0.10
**BMI, kg/m2**	27.3 (4.8)	28.4 (5.2)	27.8 (5.1)	26.6 (4.4)	27.4 (4.8)	**<0.001**
**BMI category**						**<0.001**
*<25 kg/m^2^*	786 (34%)	130 (28%)	159 (31%)	453 (38%)	44 (30%)	
*25–30 kg/m^2^*	977 (42%)	179 (39%)	205 (39%)	529 (44%)	64 (43%)	
*≥30 kg/m^2^*	571 (24%)	154 (33%)	156 (30%)	221 (18%)	40 (27%)	
**Appetite**						**0.013**
*Very good*	970 (42%)	189 (42%)	204 (40%)	531 (45%)	46 (31%)	
*Good*	851 (37%)	160 (35%)	190 (37%)	439 (37%)	62 (42%)	
*Moderate to poor*	485 (21%)	104 (23%)	120 (23%)	221 (19%)	40 (27%)	
**Healthy Eating Index, 0–100**	69.6 (12.2)	68.6 (12.5)	70.2 (11.8)	69.8 (12.3)	69.3 (11.6)	0.2
**Energy expenditure (Kcal/kg/Week)**	3.0 [0.4–9.5]	1.8 [0.1–6.8]	1.8 [0.1–7.5]	4.2 [0.7–12.0]	2.6 [0.1–9.1]	**<0.001**
**Cardiovascular disease**	628 (27%)	154 (33%)	151 (29%)	290 (24%)	33 (22%)	**<0.001**
**Hypertension**	1237 (53%)	290 (63%)	305 (59%)	568 (47%)	74 (50%)	**<0.001**
**Diabetes**	911 (39%)	212 (46%)	219 (42%)	423 (35%)	57 (39%)	**<0.001**
**Cancer**	430 (18%)	90 (19%)	97 (19%)	219 (18%)	24 (16%)	0.8
**Peripheral artery disease**	108 (4.7%)	30 (6.6%)	33 (6.5%)	37 (3.2%)	8 (5.5%)	**0.003**
**Osteoporosis**	235 (10%)	55 (12%)	57 (11%)	106 (9.0%)	17 (12%)	0.2
**Depression**	221 (9.5%)	48 (10%)	56 (11%)	100 (8.4%)	17 (11%)	0.3
**Pulmonary disease**	261 (11%)	73 (16%)	72 (14%)	105 (8.8%)	11 (7.4%)	**<0.001**
**Total prescription medications**	3.0 [1.0–5.0]	3.0 [2.0–5.0]	3.0 [1.0–5.0]	2.0 [1.0–4.0]	3.0 [1.0–4.0]	**<0.001**
**Number of hospitalizations during follow-up**						**<0.001**
*None*	1212 (52%)	168 (36%)	241 (46%)	731 (61%)	72 (49%)	
*Once*	584 (25%)	132 (29%)	139 (27%)	271 (23%)	42 (28%)	
*More than once*	538 (23%)	163 (35%)	140 (27%)	201 (17%)	34 (23%)	
**C-reactive protein (ug/mL)**	2.8 [1.2–6.2]	3.4 [1.5–7.5]	3.0 [1.2–7.1]	2.6 [1.1–5.6]	3.2 [1.2–6.3]	**<0.001**
**Interleukin-6 (pg/mL)**	2.3 [1.5–3.9]	2.8 [1.8–4.4]	2.5 [1.7–4.1]	2.0 [1.4–3.6]	2.4 [1.5–4.1]	**<0.001**
**Cystatin C (mg/dL)**	1.0 [0.9–1.1]	1.0 [0.9–1.2]	1.0 [0.9–1.1]	1.0 [0.9–1.1]	1.0 [0.9–1.1]	**<0.001**
**Creatinine (mg/dL)**	1.0 [0.9–1.2]	1.0 [0.9–1.2]	1.0 [0.9–1.2]	1.0 [0.9–1.2]	1.0 [0.9–1.1]	0.8

Note: Mean (SD), median [Q1, Q3], or frequency (%) were presented and compared using ne-way analysis of means (not assuming equal variances), Pearson’s Chi-squared test, or Kruskal–Wallis rank sum test.

**Table 3:** Top results from pathway analyses based on the 81 metabolites significantly associated with both concurrent walking ability index (WAI) and fast WAI decline and based on the 18 metabolites significantly associated with fast WAI decline only.

Pathway	Reference Metabolome Using All Metabolites in Pathway Library		Reference Metabolome Using Metabolites Measured in the Health ABC Study		Impact
	Match Status	*p*-Value	Match Status	*p*-Value	
Top pathways involving 81 consistent metabolites					
Arginine biosynthesis	3/14	0.000	3/10	0.193	0.00
Citrate cycle (TCA cycle)	3/20	0.001	3/6	0.049	0.12
Pyruvate metabolism	3/23	0.002	3/3	0.003	0.03
Nicotinate and nicotinamide metabolism	2/15	0.012	2/5	0.176	0.00
Histidine metabolism	2/16	0.013	2/6	0.240	0.00
Alanine, aspartate, and glutamate metabolism	2/28	0.038	2/9	0.432	0.23
Glyoxylate and dicarboxylate metabolism	2/32	0.049	2/8	0.369	0.03
Glycerophospholipid metabolism	2/36	0.061	2/7	0.305	0.12
Tyrosine metabolism	2/42	0.080	2/4	0.117	0.03
Ascorbate and aldarate metabolism	1/9	0.098	1/2	0.292	0.52
Top pathways involving 18 longitudinal-only metabolites					
Primary bile acid biosynthesis	3/46	0.001	3/9	0.016	0.02
Purine metabolism	3/70	0.004	3/7	0.007	0.09
One carbon pool by folate	2/26	0.007	2/7	0.076	0.04
Valine, leucine, and isoleucine biosynthesis	1/8	0.040	1/6	0.310	0.00
Taurine and hypotaurine metabolism	1/8	0.040	1/2	0.136	0.43
Pantothenate and CoA biosynthesis	1/20	0.096	1/5	0.310	0.00

## Data Availability

Data included in this study are available at NIA (https://www.nia.nih.gov/healthabc-study (accessed on 27 August 2022)).

## Abbreviations

The following abbreviations are used in this manuscript:

WAI	Walking Ability Index
TG	Triacylglycerol
DG	Diacylglycerol
LPC	Lysophosphatidylcholine
CE	Cholesterol Ester
